# Characteristics and subtypes of depressive symptoms in Chinese female breast cancer patients of different ages: a cross-sectional study

**DOI:** 10.3934/publichealth.2021055

**Published:** 2021-10-20

**Authors:** Yanyan Li, Hong Liu, Yaoyao Sun, Jie Li, Yanhong Chen, Xuan Zhang, Juan Wang, Liuliu Wu, Di Shao, Fenglin Cao

**Affiliations:** 1 Department of Nursing Psychology, School of Nursing and Rehabilitation, Cheeloo College of Medicine, Shandong University, Shandong Province, China; 2 Center for Health Management and Policy Research, Shandong University, Shandong Province, China; 3 Department of Gastroenterology, Shandong Cancer Hospital and Institute, Shandong Province, China

**Keywords:** breast cancer, depressive symptoms, latent class analysis, heterogeneity, China, age groups

## Abstract

**Purpose:**

To identify the characteristics and subtypes of depressive symptoms and explore the relationship between depressive subtypes and age among Chinese female breast cancer patients.

**Method:**

In this cross-sectional study, 566 breast cancer patients were recruited from three tertiary comprehensive hospital in Shandong Province, China through convenient sampling from April 2013 to June 2019. Depressive symptoms were measured using the Patient Health Questionnaire-9 (PHQ-9). Data analyses included descriptive analyses, latent class analysis.

**Results:**

There were significant differences in specific depressive symptoms by age group, but no significant difference in total scores on PHQ-9. The depressive subtypes were severe (Class 4), relatively severe (Class 3; with lower psychomotor agitation/retardation and suicidal ideation), moderate (Class 2; with higher psychomotor agitation/retardation and suicidal ideation), and mild depressive symptoms (Class 1). The distribution of depression subtypes is different in various age groups. In the 45–59 age groups, severe symptoms subtype showed the highest ratios (i.e. 50.3%).

**Conclusion:**

This is the first study that analyses depressive symptom characteristics and identifies depressive subtypes in Chinese women with breast cancer across ages to explore symptom heterogeneity. Our findings can contribute to identifying the mechanisms behind these relationships and developing targeted interventions for patients with specific depressive subtypes.

## 1. Introduction

In recent years improvements in the diagnosis and treatment of cancer have increased survival rates. While breast cancer, the most common type of cancer [1], has seen a significantly increasing trend in age-standardized incidence rates in Chinese women [2], the 5-year relative survival rate has increased from 73.1% to 82.0% from 2003 to 2015 [3].Owing to its untreatable nature and the common long-term exposure of patients to the illness, breast cancer tends to evoke significant psychological stress and disorders in survivors, of which depression is particularly common [4]. Two studies have shown that the prevalence of depression in cancer patients is several-fold that of the general population [5],[6], while a recent meta-analysis found that the global prevalence of depression in breast cancer patients was 32.2% [7].

Depression in female breast cancer survivors, even if it has not been properly diagnosed (e.g. the survivor has been experiencing specific depressive symptoms), may interfere with their ability to effectively cope with the disease, reduce treatment adherence, decrease quality of life, and increase the risk of recurrence and mortality [8]–[12]. Therefore, attention should be paid to female breast cancer survivors with depressive symptoms, regardless of a clinical diagnosis.

Women in different age groups may face various challenges when coping with breast cancer, resulting in different depressive symptoms by age [13],[14]. For example, younger female breast cancer patients may experience psychological stress from life-stage-related needs that occur at a younger age (e.g. employment, childcare) [15] and from perceptions regarding the fact that diagnosis and treatment may cause the partial loss of their female identity, infertility, premature menopause, and sexual dysfunction [16]–[19]. Middle-aged breast cancer female survivors normally face psychological stress from having to take care of their parents—usually in later adulthood—and from considering the potential impact of breast cancer on their children [13]. Older female adults who incur normal ageing and comorbidities (e.g. chronic diseases, age-related diseases, and geriatric problems) tend to be affected by cancer symptoms, leading to the further decline of their bodily functions [20]. Therefore, various age-related sources of stress may lead to inconsistency and heterogeneity when comparing the depressive symptoms of female breast cancer patients by age.

A few studies have analysed depressive symptoms in female breast cancer patients of different ages, but their sample had a limited age range (e.g. under 35 [21] or over 60 [22]), or their findings only compared the total scores for depressive symptoms between two age groups (e.g. ≤45 vs. 55–70 [23], ≤50 vs. >50 [24], or 18–39 vs. >39 [14]). A prior study corroborated this and concluded that, although research on the topic provided valuable evidence, researchers tend to only use the total or cut-off score of the depressive symptom scale, which conceals the heterogeneity of depressive symptoms in female cancer patients [25]. For example, even if two participants have the same total score on the depressive symptom scale, their scores may be based on different symptoms (e.g., suicidal ideation versus fatigue).

Thus, we considered latent class analysis (LCA) appropriate to explore the subtypes of breast cancer related depression while considering the potential correlation between different depressive symptoms. The LCA is a type of person-centred analytical method that focuses on distinguishing a heterogeneous population that has co-occurring symptoms to reveal potential symptomatic heterogeneity [26]. It may be helpful when trying to objectively identify heterogeneous depressive subtypes in clinical practice; doing so may provide clinicians with more information on how to develop effective prevention and intervention programmes for female breast cancer patients with different depressive subtypes.

To the best of our knowledge, no prior study to date has detailed the characteristics of depressive symptoms by multiple age groups or used depressive subtypes to explore depressive symptom heterogeneity among female breast cancer patients. Thus, this study aimed to (1) describe the characteristics of depressive symptoms, (2) identify depressive subtypes, and (3) explore the relationship between depressive subtypes and age in Chinese female breast cancer patients.

## 2. Methods

### 2.1. Study design

This study applied a cross-sectional design.

### 2.2. Participants

Using the convenient sampling method, we recruited female breast cancer patients by contacting three tertiary comprehensive hospitals in Shandong Province, China from April 2013 through June 2019. The inclusion criteria were as follows: (1) at least 18 years of age; (2) has been diagnosed with breast cancer; and (3) able to understand and independently answer the questionnaire. The initial sample of 573 potential participants was reduced to 566 after excluding those who did not complete a Patient Health Questionnaire-9 (PHQ-9) or indicate age data.

First, eligible patients were identified using medical records from cancer wards. Then, they were asked by the investigators, who were graduate students in the psychology department, whether or not they were willing to participate in the study. Participants who agreed gave their written informed consent before completing the questionnaire. After receiving approval from the cancer ward, we collected cancer stage data from patients' medical records. Other questionnaire items were answered through a self-report.

All procedures followed were in accordance with the ethical standards of the responsible committee on human experimentation (institutional and national) and the Helsinki Declaration of 1975, as revised in 2000. The survey formed part of a research project on the mental health status of cancer patients, which was approved by the ethics committee of the School of Nursing at the Shandong University (Ethical Approval Number: 2016-R020).

### 2.3. Measures

We measured depressive symptoms using the Chinese version of the 9-item PHQ-9, which the American Society of Clinical Oncology recommends for the evaluation of depressive symptoms in cancer patients [27]. The PHQ-9 assesses the symptoms of participants in the 2 weeks prior to the questionnaire application [28]. It is rated on a 4-point scale ranging from 0 (not at all) to 3 (nearly every day). The nine items assess nine depressive symptoms based on the diagnostic criteria for major depressive disorder described in the DSM-IV. Participants' total score in this measure represents depressive severity, categorised as minimal (0–4), mild (5–9), moderate (10–14), and moderately severe/severe depressive symptoms (≥15). The Cronbach's alpha in this study was 0.824.

The covariate variables were age (≤35, 36–44, 45–59, and ≥60 years old), race (Han/other minorities), marital status (single/partnered), residential area (urban/rural), education level (high school or lower/above high school), occupation (retired/unemployed/employed), mode of payment of medical care (own expense/health insurance), religion (religious/not religious), and cancer stage (0/I/II/III/IV).

### 2.4. Statistical analysis

All descriptive analyses were performed using SPSS (version 26.0). This study compared participants' age by sociodemographic, clinical (e.g. cancer stage), and depressive characteristics (i.e. participants' mean scores for each symptom and depressive severity category based on the total PHQ-9 score).

We used a nonparametric analysis to examine the differences among the nine depressive symptoms and the depressive severity by age group. Univariate analyses, including ANOVA and Chi-square test, were used to determine differences in depressive subtypes by socio-demographic and clinical characteristics. Subsequently, analysis of variance and post-hoc tests were used to assess the differences in depressive subtypes by the nine depressive symptoms. We set the statistical significance for all analyses at *P* < 0.05.

We used LCA to identify the differences in depressive subtypes among female breast cancer patients, namely, whether or not there were homogeneous clusters in heterogeneous groups. We recorded the nine symptoms measured in the PHQ-9 as binary variables (1 = have depressive symptoms vs. 0 = no depressive symptoms) and included them in the model.

We tested goodness of fit for a series of models (i.e. 1–6 classes) using Mplus (version 7.4). By analysing a combination of statistical indicators—including Akaike information criterion, Bayesian information criterion, sample size adjustment BIC, Vuong-Lo-Mendell-Rubin likelihood ratio test, bootstrap likelihood ratio test, and clinical interpretability [29]—we deemed that the four-class model showed optimal fit to the data.

## 3. Results

### 3.1. Participants' characteristics

The participants had an average age of 45.6 (SD = 11.5) years; most were married (93.3%); unemployed (42.4%); and had medical insurance (95.6%). Approximately half lived in urban areas (57.6%). A majority had stage II cancer (41.3%), followed by stage IV (20.8%), III (20.7%), I (13.8%), then 0 (3.4%). The four age groups differed in many other sociodemographic and clinical characteristics, which are described in detail in **Table 1**.

**Table 1.** Sociodemographic and clinical characteristics of the total sample and different age groups (N = 566).

Variables	Total (n = 566)	≤35 years old (n = 95)	36–44 years old (n = 223)	45–59 years old (n = 151)	≥60 years old (n = 97)	*P*
Age, *x(SD)/n(%)*	45.6 (11.5)	95 (16.8%)	266 (47.0%)	108 (19.1%)	97 (17.1%)	
Marriage						0.022
Single	38 (6.7%)	10 (10.5%)	15 (6.7%)	3 (2.0%)	10 (10.3%)	
With partner	528 (93.3%)	85 (89.5%)	208 (93.3%)	148 (98.0%)	87 (89.7%)	
Residence						0.112
Urban area	326 (57.6%)	55 (57.9%)	139 (61.7%)	75 (49.7%)	57 (58.8%)	
Rural area	240 (42.4%)	40 (42.1%)	84 (38.3%)	76 (50.3%)	40 (41.2%)	
Education						<0.001
≤High school	321 (56.7%)	31 (32.6%)	113 (53.0%)	101 (66.9%)	76 (78.4%)	
>High school	245 (43.3%)	64 (67.4%)	110 (47.0%)	50 (33.1%)	21 (21.6%)	
Occupation ^†^						<0.001
Retirement	59 (10.4%)	1 (1.3%)	0 (0.0%)	13 (8.6%)	45 (47.9%)	
Unemployed	235 (41.5%)	28 (35.4%)	88 (39.5%)	75 (49.7%)	44 (46.8%)	
Employed	207 (36.6%)	50 (63.3%)	112 (50.2%)	40 (26.5%)	5 (5.3%)	
Payment ^†^						0.390
Own expense	15 (2.7%)	2 (2.1%)	9 (4.0%)	3 (2.0%)	1 (1.0%)	
With health care	541 (95.6%)	93 (97.9%)	210 (94.2%)	144 (95.4%)	94 (99.0%)	
Religion	77 (13.6%)	14 (14.7%)	36 (16.1%)	16 (10.6%)	11 (11.3%)	0.405
Race (Han)	557 (98.4%)	93 (97.9%)	265 (99.6%)	103 (95.4%)	96 (99.0%)	0.167
Cancer stage						<0.001
Stage 0	19 (3.4%)	2 (2.1%)	8 (3.6%)	1 (0.7%)	8 (8.2%)	
Stage Ⅰ	78 (13.8%)	13 (13.7%)	35 (15.7%)	9 (6.0%)	21 (21.7%)	
Stage Ⅱ	234 (41.3%)	41 (43.2%)	111 (49.8%)	38 (25.2%)	44 (45.4%)	
Stage Ⅲ	117 (20.7%)	23 (24.2%)	45 (20.2%)	36 (23.8%)	13 (13.4%)	
Stage Ⅳ	118 (20.8%)	16 (16.8%)	24 (10.8%)	67 (44.4%)	11 (11.3%)	

Note: ^†^ Indicates that the numbers/percentages may not add up to the total, due to missing data.

### 3.2. Differences in depressive symptoms among female breast cancer patients by age

**Figure 1.**
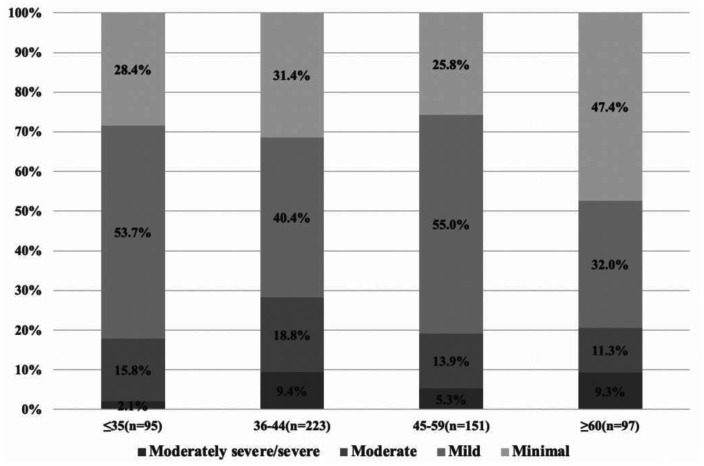
The severity of depressive symptoms varies among breast cancer patients of different ages according to the PHQ-9 cutoff scores. Minimal depression (PHQ-9 score 0–4), mild (5–9), moderate (10–14) and moderately severe/severe (≥15). PHQ-9, Patient Health Questionnaire-9.

Of all participants, 22.8% had moderate to severe depressive symptoms (i.e. PHQ-9 scores ≥10). The percentage for moderately severe/severe depressive symptoms (i.e. PHQ-9 scores ≥15) was 2.1% for participants aged ≥35, 9.4% for those aged 36–44, 5.3% for those aged 45–59, and 9.3% for those aged ≥60 (**Figure 1**).

**Table 2.** Characteristics of depression in different age groups (N = 566).

M (Q_L_;Q_U_)/n(%)	Total (n = 566)	≤35 years	36–44 years	45–59 years	≥60 years	*P*
Anhedonia	1.0 (0.0;1.0)	1.0 (0.0;1.0)	1.0 (0.0;1.0)	1.0 (1.0;1.0)	1.0 (0.0;1.0)	0.095
Sadness	1.0 (0.0;1.0)	1.0 (0.0;1.0)	1.0 (0.0;1.0)	1.0 (1.0;2.0)	1.0 (0.0;1.0)	0.001
Sleep disturbances	1.0 (1.0;1.0)	1.0 (1.0;1.0)	1.0 (0.0;1.0)	1.0 (1.0;2.0)	1.0 (0.0;2.0)	0.328
Fatigue	1.0 (1.0;1.0)	1.0 (1.0;1.0)	1.0 (1.0;2.0)	1.0 (1.0;1.0)	1.0 (0.0;1.0)	0.286
Appetite disturbances	1.0 (0.0;1.0)	1.0 (0.0;1.0)	1.0 (0.0;1.0)	1.0 (1.0;1.0)	1.0 (0.0;1.0)	0.178
Guilt or worthlessness	1.0 (0.0;1.0)	1.0 (0.0;1.0)	1.0 (0.0;1.0)	1.0 (0.0;1.0)	0.0 (0.0;1.0)	0.064
Poor concentration	0.0 (0.0;1.0)	1.0 (0.0;1.0)	1.0 (0.0;1.0)	0.0 (0.0;1.0)	0.0 (0.0;1.0)	0.003
Psychomotor agitation or retardation	0.0 (0.0;1.0)	0.0 (0.0;1.0)	0.0 (0.0;1.0)	0.0 (0.0;1.0)	0.0 (0.0;1.0)	0.003
Suicidal ideation	0.0 (0.0;0.25)	0.0 (0.0;0.0)	0.0 (0.0;1.0)	0.0 (0.0;0.0)	0.0 (0.0;0.5)	<0.001
PHQ total	7.0 (4.0;9.0)	7.0 (4.0;9.0)	7.0 (4.0;10.0)	7.0 (4.0;9.0)	5.0 (2.0;9.0)	0.034
PHQ average	0.8 (0.4;1.0)	0.8 (0.4;1.0)	0.8 (0.4;1.1)	0.8 (0.4;1.0)	0.6 (0.2;1.0)	0.034
PHQ-9 (≥5)	384 (67.8%)	68 (71.6%)	153 (68.6%)	112 (74.2%)	51 (52.6%)	0.003
PHQ (≥10)	129 (22.8%)	17 (17.9%)	63 (28.3%)	29 (19.2%)	20 (20.6%)	0.092
PHQ degree						0.001
Minimal	182 (32.2%)	27 (28.4%)	70 (31.4%)	39 (25.8%)	46 (47.4%)	
Mild	255 (45.1%)	51 (53.7%)	90 (40.4%)	83 (55.0%)	31 (32.0%)	
Moderate	89 (15.7%)	15 (15.8%)	42 (18.8%)	21 (13.9%)	11 (11.3%)	
Moderately severe/severe	40 (7.1%)	2 (2.1%)	21 (9.4%)	8 (5.3%)	9 (9.3%)	

There were significant differences were found for the following symptoms: sadness, poor concentration, psychomotor agitation/retardation, and suicidal ideation. In addition, participants of different age groups differed in the severity of depressive symptoms (see **Table 2**).

### 3.3. Heterogeneity and depressive subtypes in female breast cancer patients

**Figure 2.**
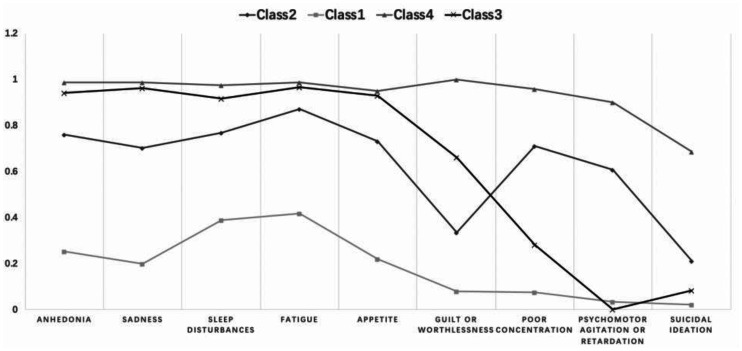
The five-class model and probability of nine depressive symptoms within each class (n = 566). Class 4: severe symptoms group; Class 3: relatively severe group (with lower concentration, psychomotor agitation/retardation and suicidal ideation); Class 2: moderate symptoms group (with higher poor concentration, psychomotor agitation/retardation and suicidal ideation); Class 1: mild symptoms group.

Considering goodness of fit and clinical interpretability, we determined four depressive subtypes: Class 1, 2, 3, and 4; these accounted for 27%, 16%, 30%, and 27% of all participants, respectively (**Table 3**). **Figure 2** and **Table 4** show the probability and descriptive data of participants' scores for the nine depressive symptoms in the PHQ-9 by depressive subtype. Class 4 represented the highest probability (i.e. probability of experiencing a symptom) and score for all nine symptoms; thus, it was named the severe symptoms group; Class 3 represented a relatively high probability and score for all symptoms—except for psychomotor agitation/retardation and suicidal ideation, which showed lower levels—and thus, it was named the relatively severe symptoms group; Class 2 represented a medium probability and score for all nine symptoms—except for psychomotor agitation/retardation and suicidal ideation, which showed higher levels—and thus, it was named the moderate symptoms group; and Class 1 represented the lowest probability and score for all nine symptoms—except for psychomotor agitation/retardation, which showed higher levels—and therefore, it was named the mild symptoms group. There were significant differences between these four classes and the nine depressive symptoms assessed using the PHQ-9 (**Table 4**).

**Table 3.** Model fit indices derived from latent class analysis on models with 1–6 classes.

Model	K	Log (L)	AIC	BIC	aBIC	entropy	LMR	BLRT	Class probability
1	9	−3096.85	6211.70	6250.75	6222.18				
2	19	−2540.85	5119.70	5202.14	5141.82	0.86	0.00	0.00	368/198(0.65/0.35)
3	29	−2441.40	4940.80	5066.62	4974.56	0.82	0.00	0.00	152/259/155(0.27/0.46/0.27)
4	39	−2402.15	4882.30	5051.51	4927.70	0.78	0.02	0.00	155/91/170/150(0.27/0.16/0.30/0.27)
5	49	−2368.00	4833.98	5046.57	4891.02	0.80	0.002	0.00	49/141/148/130/98(0.09/0.25/0.26/0.23/0.17)
6	59	−2354.61	4827.22	5083.20	4895.90	0.81	0.46	0.09	107/38/146/52/77/146(0.19/0.07/0.26/0.09/0.14/0.26)

Note: Abbreviations: AIC, Akaike information criterion; BIC, Bayesian information criterion; aBIC, sample size adjusted BIC; LMR, Vuong-Lo-Mendell-Rubin likelihood ratio test. BLRT, bootstrapped likelihood ratio test; Bold values indicates that a four-class model was determined as optimal one.

**Table 4.** The descriptive scores of nine depressive symptoms, *M(SD)*.

Items	Class 4 (n = 150)	Class 3 (n = 170)	Class 2 (n = 91)	Class 1 (n = 155)	F	Posthoc comparisons
Anhedonia	1.40 (0.63)	1.19 (0.57)	0.86 (0.68)	0.26 (0.52)	109.39*	c4>c3>c2>c1
Sadness	1.41 (0.65)	1.42 (1.62)	0.75 (0.59)	0.19 (0.43)	53.55*	c3≈c4>c2>c1
Sleep disturbances	1.49 (0.70)	1.31 (1.03)	1.04 (0.87)	0.43 (0.62)	49.75*	c4>c3>c2>c1
Fatigue	1.59 (0.71)	1.20 (0.53)	1.13 (0.70)	0.49 (0.70)	73.87*	c4>c3≈c2>c1
Appetite disturbances	1.37 (0.68)	1.06 (0.81)	0.88 (0.73)	0.21 (0.43)	81.11*	c4>c3>c2>c1
Guilt or worthlessness	1.50 (0.67)	0.76 (0.67)	0.34 (0.67)	0.07 (0.26)	163.33*	c4>c3>c2>c1
Poor concentration	1.35 (0.64)	0.34 (0.61)	1.01 (0.71)	0.07 (0.26)	159.56*	c4>c2>c3>c1
Psychomotor agitation or retardation	1.30 (0.69)	0.00 (0.00)	0.81 (0.65)	0.04 (0.22)	288.45*	c4>c2>c1≈c3
Suicidal ideation	0.89 (0.73)	0.07 (0.30)	0.31 (0.61)	0.03 (0.16)	102.77*	c4>c2>c3≈c1
PHQ-9 total score	12.29 (3.85)	7.36 (2.81)	7.13 (2.29)	1.79 (1.47)	362.34*	c4>c3≈c2>c1

Note: *P < 0.01. Class 4: severe symptoms group; Class 3: relatively severe group (with lower concentration, psychomotor agitation/retardation and suicidal ideation); Class 2: moderate symptoms group (with higher poor concentration, psychomotor agitation/retardation and suicidal ideation); Class 1: mild symptoms group.

**Figure 3.**
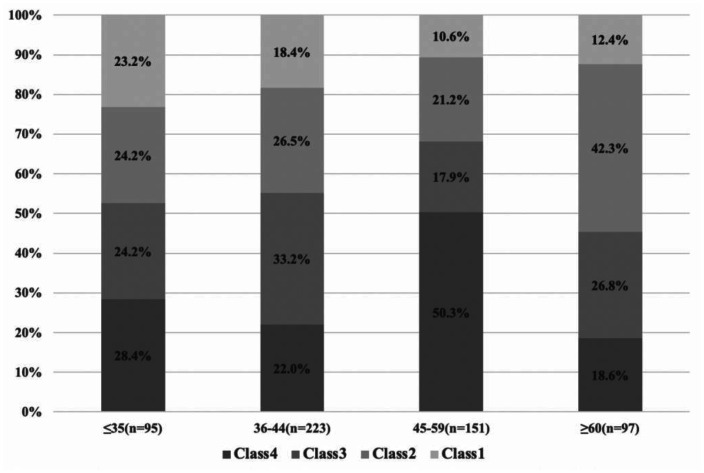
The subtypes distribution in the four stage of age. Class 4: severe symptoms group; Class 3: relatively severe group (with lower concentration, psychomotor agitation/retardation and suicidal ideation); Class 2: moderate symptoms group (with higher poor concentration, psychomotor agitation/retardation and suicidal ideation); Class 1: mild symptoms group.

Participants' depressive subtype distribution by age is presented in **Figure 3**. In the ≤35 age group, all four classes showed similar ratios (Class 1–4 were 28.4%, 24.2%, 24.2%, and 23.2%, respectively). In the 36–44 age group, 33.2% of the participants were categorized in Class 3, which was the highest proportion. In the 45–59 age groups, Class 4 showed the highest ratios (i.e. 50.3%). In the ≥60 age group, more than 50 percent of participants were categorized in the milder depressive subtypes, with Class 1 and 2 accounting for 12.4% percent and 42.3%, respectively.

## 4. Discussion

This study aimed to analyse the depressive characteristics of breast cancer patients in different adult age groups. We were able to identify four latent depressive subtypes, and their distribution differed by age group. To the best of our knowledge, this is the first study to analyse and use potential depressive subtypes to explore the heterogeneity of depressive symptoms across various adult age groups in female breast cancer patients.

The incidence of moderate to severe depressive symptoms in our sample was 22.8%; this number was similar to that reported in a previous study, which showed that such incidence in female breast cancer patients was 2–3 times higher than that in the general population [14]. Additionally, our analyses highlighted differences in single-symptom expression by age—specifically, such differences were observed for sadness, poor concentration, psychomotor agitation/retardation, and suicidal ideation. This result is supported by that of a previous study, which reported on individuals' symptomatology heterogeneity [25]. Another study showed that the age-related difference in depressive symptoms among female cancer patients is due to the impact of cancer and its treatment on specific areas of women's life (e.g. work, sex, and entertainment) [15]. Therefore, stakeholders involved in diminishing the risk of depression in breast cancer patients should focus on depressive symptom differences by age. Moreover, in-depth analyses on the causes of such age-related differences must be conducted and interventions aimed at groups characterised by specific depressive subtypes must be applied to reduce the risk of depression.

We were able to identify four depressive subtypes: severe (Class 4), relatively severe (Class 3), moderate (Class 2), and mild depressive symptoms (Class 1). Differences in the severe, moderate, and mild groups were mainly because of depressive symptom severity, whereas the relatively severe group differed from the other three primarily owing to the presence of severe physio-somatic symptoms alongside lower psychomotor agitation/retardation and suicidal ideation. This suggests that, upon the application of LCA, both symptom characteristics and severity were important variables in determining depressive subtypes. Our results are somewhat consistent with those of previous studies showing symptomatic severity to be a crucial discriminating aspect of depressive subtypes [30],[31]. The relatively severe group with lower psychomotor agitation/retardation and suicidal ideation was discovered in this research, and it was also the most common subtype in our sample. A study that promoted a factorial analysis on the items of the PHQ-9 pointed out that the items on poor concentration and psychomotor agitation/retardation may neither belong to an affective-cognitive nor a somatic component [32]. This item contains two contradictory characteristics of psychological and cannot be clearly classified as one of the factors, which also confirms the heterogeneity of depressive symptoms. Previous studies have suggested that suicidal ideation in breast cancer patients is particularly linked to genetic characteristics (brain-derived neurotrophic factor methylation, BDNF *met* allele) [33]. Along with another study [34], these findings and our results suggest that depressive symptoms do not have a single structure but are comprised of different subtypes that seem to have different pathophysiological basis.

The latent depressive subtypes we observed showed different distributions by age group. Specifically, patients aged 45–59 were more likely to have severe depressive subtype; those aged 36–44 were more likely to have relatively severe depressive subtype; those over 60 were more likely to have moderate symptoms group; and those under 35 were more likely to have mild depressive subtype. After a literature review, it is evident why female breast cancer patients aged 36 and over showed a greater tendency to experience relatively severe depressive symptoms. A study showed that perimenopausal women over 40 may suffer from poorer sleep quality and greater mood problems owing to fluctuating hormone levels, both of which are also depressive symptoms [35]. Another study revealed that breast cancer treatment may lead to menopause in women, while the stress caused by cancer and its treatment may influence depressive symptom severity [36]. When combined with the social responsibilities that female breast cancer survivors tend to have, such as caring for parents and children, the situation can lead them to experience the most severe depressive symptoms [13]. Therefore, stakeholders in the well-being of female breast cancer patients should place greater emphasis on their psychological status based on age. In clinical practice, the different depressive subtypes—and their relationships with specific age groups—described in this study may help stakeholders (e.g. physicians, psychologists, nurses) more accurately identify groups with similar symptoms across the cancer population. Greater accuracy could thereby facilitate the development and application of appropriate group interventions aimed at dealing with similar depressive symptoms.

Despite the contributions highlighted above, this study had several limitations. First, the reliability and validity of individual symptom measurement tools—including those of the PHQ-9, which was utilised in this study—remain imprecise. Nonetheless, one advantage of the PHQ-9 is that all entries have the same response categories, which theoretically does not affect the comparability between different symptoms [33]. Future research should investigate scales for specific symptoms (e.g. suicidal ideation), such as the Suicide Severity Scale to assess suicidal behaviour [37]. Second, although the LCA assigns individuals to subtypes according to probability and evaluates the goodness of fit of different models based on statistical criteria, one study has shown that subjectivity in this procedure still exists [29]; therefore, we cannot exclude type I errors (i.e. false positives). Third, we applied convenient sampling and utilised a cross-sectional design, which are methodologies that limit the generalizability of our findings; thus, future research should consider larger populations, stratified random sampling, and a longitudinal design when analysing depressive symptoms in female breast cancer patients.

## 5. Conclusions

This study described the characteristics of depressive symptoms in Chinese female breast cancer patients across different ages and identified four depressive subtypes. Our results support the heterogeneity of depressive symptoms; thus, we provide data on how to identify individual symptoms in different age groups and patients with similar symptoms characteristics. We hope that this study helps in identifying the potential mechanisms behind these relationships and develop targeted interventions for patients with a specific depressive subtype.
